# POM/EVA Blends with Future Utility in Fused Deposition Modeling

**DOI:** 10.3390/ma13132912

**Published:** 2020-06-29

**Authors:** Mateusz Galeja, Klaudiusz Wypiór, Jan Wachowicz, Przemysław Kędzierski, Aleksander Hejna, Mariusz Marć, Krzysztof Klewicz, Jadwiga Gabor, Hubert Okła, Andrzej Szymon Swinarew

**Affiliations:** 1 Department of Material Engineering, Central Mining Institute, Pl. Gwarków 1, 40-166 Katowice, Poland; maxgalley@gmail.com (M.G.); kwypior@gig.eu (K.W.); jwachowicz@gig.eu (J.W.); 2 Department of Acoustics, Electronics and IT Solutions, Central Mining Institute, Pl. Gwarków 1, 40-166 Katowice, Poland; pkedzierski@gig.eu; 3 Department of Polymer Technology, Gdańsk University of Technology, Narutowicza 11/12, 80-233 Gdańsk, Poland; 4 Department of Analytical Chemistry, Gdańsk University of Technology, Narutowicza 11/12, 80-233 Gdańsk, Poland; mariusz.marc@pg.edu.pl (M.M.); krzysztof.klewicz@pg.edu.pl (K.K.); 5 Faculty of Science and Technology, University of Silesia in Katowice, 41-500 Chorzów, Poland; jadwiga.gabor@us.edu.pl (J.G.); hubert.okla@us.edu.pl (H.O.); andrzej.swinarew@us.edu.pl (A.S.S.); 6 Institute of Sport Science, The Jerzy Kukuczka Academy of Physical Education, 40-065 Katowice, Poland

**Keywords:** polyoxymethylene, ethylene-vinyl acetate, polymer blends, 3D printing, warping, volatile organic compounds

## Abstract

Polyoxymethylene (POM) is one of the most popular thermoplastic polymers used in the industry. Therefore, the interest in its potential applications in rapid prototyping is understandable. Nevertheless, its low dimensional stability causes the warping of 3D prints, limiting its applications. This research aimed to evaluate the effects of POM modification with ethylene-vinyl acetate (EVA) (2.5, 5.0, and 7.5 wt.%) on its processing (by melt flow index), structure (by X-ray microcomputed tomography), and properties (by static tensile tests, surface resistance, contact angle measurements, differential scanning calorimetry, and thermogravimetric analysis), as well as very rarely analyzed emissions of volatile organic compounds (VOCs) (by headspace analysis). Performed modifications decreased stiffness and strength of the material, simultaneously enhancing its ductility, which simultaneously increased the toughness even by more than 50% for 7.5 wt.% EVA loading. Such an effect was related to an improved linear flow rate resulting in a lack of defects inside the samples. The decrease of the melting temperature and the slight increase of thermal stability after the addition of EVA broadened the processing window for 3D printing. The 3D printing trials on two different printers showed that the addition of EVA copolymer increased the possibility of a successful print without defects, giving space for further development.

## 1. Introduction

Three-dimensional (3D) printing, also known as additive manufacturing, is described as the incremental digital technology that allows creating physical objects by successive addition of material. This manufacturing technology has originated from three-dimensional (3D) layer-by-layer fabrication of computer-aided design (CAD) drawing [1].

Three-dimensional printing is used worldwide to produce customizable designs and small series production in many fields, including the automotive and aerospace industries [1,2]. Before the final release or commercialization of tested products, prototypes printed with rapid prototyping technology can be useful in project design validation, in assessing the resolution quality, and in material stability checks. This gives significant advantages in future processing, helps streamline industrial development with small series rapid retooling individualization, and also influences the end price of the prototype with linear costs that depend mostly on the number of pieces produced [2,3].

Well-known and widely used extrusion additive manufacturing (AM), commonly known as fused deposition modeling (FDM), is a technique based on layer manufacturing technology with principles of surface chemistry and thermal energy used [4]. A wide range of parameters makes this process complex to understand and control. Such an effect is related to the interdependence between the physicochemical properties of the final product [5], which, according to Cuan-Urquizo et al. [6], results in the anisotropic nature of this technology. Plane of deposition (PTB) properties are significantly different from prints made along with the stacking direction (ST). Multiple works conducted on fused filament fabrication (FFF) raised the topic of raster pattern and angle influence on the mechanical properties of specimens [7,8].

The printing process starts with heating the head to reach the temperature of the material melting point (usually lower than 232 °C) [9]. The deposition temperature has a significant impact on the layer-to-bed and layer-to-layer adhesion quality. This is due to solidification through physical processes, such as crystallization and glass transition of thermoplastics, and solidification through chemical reactions, including thermoset crosslinking [10]. Limitations characterize this part due to the usage of high temperatures, which may lead to potential thermal degradation of the used material, shrinkage, adhesion problems, and overall low quality of the printed product. Difficulties related to printing parameters limit the number of thermoplastic polymers used in the industry [5].

The increasing popularity of FDM in low-volume applications due to faster fabrication and design flexibility is strictly connected to a wide range of commodity thermoplastics, including polylactic acid (PLA), acrylonitrile butadiene styrene (ABS), polyether ether ketone (PEEK), polyetherimide (PEI) [11], polyamide 66 (PA66) [12], polyvinyl alcohol (PVA), poly(ε-caprolactone) (PCL), and ethylene-vinyl acetate (EVA) [5], and the amount of research on new materials.

Polyoxymethylene, also known as acetal, polyacetal, and polyformaldehyde, is an engineering thermoplastic with a semicrystalline polymeric structure. Low friction and wear characteristics linked with mechanical properties and chemical resistance to most solvents, chemicals, and fuels give this material a wide range of applications. Most of the POM resins are used in the automotive, electrical, and electronic parts industries [13]. With 330 thousand tons used in automotive plastic components markets in 2014, POM was the sixth most used thermoplastic, right behind the 370 thousand tons reported for polycarbonates (PC) [14]. Although POM is traditionally processed mostly by injection molding, extrusion, and subtractive manufacturing (machining), by taking into account processing parameters (extrusion around 250 °C), it seems to be a perfect match for AM technologies [15].

Despite the overall difficulties with the self-lubricating nature of POM resulting in poor adhesion of the first-printed layer to the printing table, in addition to an excessively heated base temperature exceeding 130 °C, some companies (such as Gizmo Dorks, Actifil3D, Apium, and FFF World) offer their own FDM acetal filaments [15]. The other well-known problem is the shrinkage of semicrystalline resins during cooling. In the case of POM resin, values of shrinkage are around 1.85–2.35, 1.85–2.50, and 0.05–0.25 for shrinkage parallel (RL) and orthogonal (RT), to the injection flow, and differential shrinkage (RD), respectively. These parameters are calculated according to Formulas (1)–(3) [16]:

RL = ((L_n_ − L_m_))/L_n_ × 100 (1)

RT = ((T_n_ − T_m_))/T_n_ × 100 (2)

RD = RT − RL(3)

where L_n_ is the part nominal length parallel to the injection flow, L_m_ is the real part measure parallel to the injection flow, T_n_ is the part nominal length orthogonal to the injection flow, and T_m_ is the real part measure orthogonal to the injection flow.

This information set, taking into consideration the dimensional stability of POM (60–70%) compared to ABS (85–95%), allows the conclusion that 3D printing with this material will most likely result in warping without strong adhesive or high printing bed temperature. This deformation issue was raised by many investigators [17,18,19,20], with some prevention method outcomes including a properly adjusted extruder temperature and usage of a heated build plate [17,21].

The analysis of the knowledge about polyoxymethylene in the injection molding industry, problems with 3D printing of this material, and a recent study performed by Liang [22] directed us to the modification of POM with EVA copolymer. The present study aimed to find the optimum addition and combination of thermoplastic polymer EVA to semicrystalline resin POM that may lead to improved 3D printability of this construction material. The incorporation of EVA was aimed at lowering the above-mentioned relatively high shrinkage of the POM, which should reduce the warping of the 3D prints. This effect was expected based on the higher flexibility of EVA and lower values of shrinkage. Extrusion of EVA polymer at weight fractions of 2.5, 5.0, and 7.5 wt.% allowed to systematically explore the effect of its addition on various parameters of the acetal base.

## 2. Materials and Methods

### 2.1. Materials

Polyoxymethylene copolymer Tarnoform 300 supplied by Grupa Azoty S.A. (Tarnów, Poland) was used as a model thermoplastic resin. It was characterized by a density of 1.41 g/cm^3^. Ethylene-vinyl acetate (EVA) resin Escorene Ultra FL00218 supplied by ExxonMobil Chemical (Houston, TX, USA) was used as a modifier for POM. It was characterized by a density of 0.94 g/cm^3^.

### 2.2. Sample Preparation

Materials were stored in a laboratory dryer for 8 h at the temperature of 60 °C, then samples were prepared using a Leistritz ZSE 27 HP co-rotating twin-screw extruder (L/D ratio = 44) equipped with a Brabender gravimetric dosing system with an accuracy of 0.1 wt.%. Extrusion of four samples of 5 kg each was performed at specified extrusion parameters with ten heated zones and two nozzle zones with monitored work parameters, as shown in Table 1.

**Figure 1:**
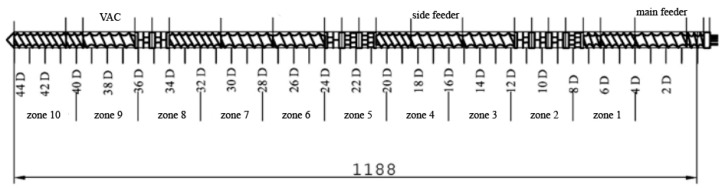
Screw configuration applied for the preparation of analyzed samples.

The screw configuration applied during sample preparation is shown in Figure 1. The following kneading segments were used in the intensive plasticizing zone: KB 5-2-30-30°, KB 5-2-30-60°, KB 5-2-30-90°. The following kneading segments were used in the first mixing zone: KB 5-2-30-30°, KB 5-2-30-60°. The second mixing zone used the following kneading segments: KB 5-2-30-60°, KB 5-2-30-90°. KB 5 indicates five blocks of kneading discs with staggering angles of 30°, 60°, and 90° in the right direction of inclination of the apparent helix of subsequent disc crests [23]. The main feed zone is responsible for the initial conveying of the main material; zone 1 is responsible for conveying and is the reaction zone; in zone 2, there is a first kneading of material; zone 3 is for transporting plasticized material to the zone of the side feeder, where the reaction zone and second kneading zones (4 and 5) allow the materials to be mixed. The steps without the side feeder are replaced in the next segments (6–8) of the twin-screw extruder. In zone 9, the vacuum pump (VAC) helps with degassing mixed materials to get a filament without air bubbles after melt pumping occurs in zone 10.

POM was dosed into the main feeder at the beginning of the extrusion barrel, while EVA was introduced by the side-feed on zone 5 with a screw speed of 200 rpm at a temperature of 87 °C. EVA was added in the amounts of 2.5, 5.0, and 7.5 wt.%. The addition of EVA was aimed at softening the polymer and reducing the warping effect of 3D-printed specimens by lowering the POM shrinkage factor by 2.0–3.5% with 1–3% of additive material. According to Uthaman et al. [24], the addition of EVA beyond 7.5% decreases the impact strength due to reduced adhesion between components. Furthermore, this study aimed to examine the effects of addition on the overall physical properties of POM.

The nozzle used in extruder allowed the production of a 5 mm filament, which was drawn by a modified single-blade Schear granulator. Adjustment of the speed of the receiving system with a dismantled cutting system allowed us to obtain a size of 2.85 ± 0.1 mm. The filament was examined using an electronic measuring caliper. The material that did not meet the printer requirement was later granulated and used in the injection molding part. The granulated POM/EVA blends were dried before the extrusion experiments at 70 °C for 8 h.

An Arburg Allrounder 270-210-500 screw injection molding machine equipped with the Pass Controller injection control system (Primus AG, Binningen, Switzerland) was used to prepare ISO-527-2-1A dogbone samples. The parameters of the injection process are summarized in Table 2.

### 2.3. Measurements

The emission studies, with results expressed by the total volatile organic compounds (TVOCs) parameter, were investigated using the microscale stationary emission chamber system (Markes International Microchamber/Thermal Extractor µ-CTE250, Markes International Ltd, Llantrisant, UK) equipped with four similar stainless steel chambers, each with a volume of 114 cm^3^. Detailed information about the microchamber characteristics and working parameters has been published elsewhere [25,26]. The sample (average mass of a sample was 1.024 ± 0.023 g) was firstly placed on a Petri glass dish, weighed, and then installed inside the chamber to prevent the potential chamber contamination. Next, the chambers were closed, and the Tenax TA stainless steel tube was installed to collect the volatile organic compounds from the gaseous phase emitted from the studied samples. The following sampling conditions were set up: sample treatment time of 30 or 60 min, nitrogen flow rate of 25 mL·min^−1^, and treatment temperature of 100 °C. After the sampling process, the stainless steel tubes were removed and tightly sealed. To extract the analytes from the applied sorption medium, the thermal desorption (TD) technique (Mareks Int, Unity 2) was used. The separation and TVOCs parameter determination were performed with the use of gas chromatography (Agilent Technologies 7820A, Agilent, Santa Clara, CA, USA) combined with a flame ionization detector (FID). The Tenax TA sorption tubes were desorbed at the temperature of 285 °C by 12 min, and analytes were transported to the microtrap (0 °C). Liberated from the microtrap, chemical compounds (at 300 °C for 5 min) were transported by the helium flow rate (2.0 mL·min^−1^) to the gas chromatography (GC) capillary column (J&W, DB-1, 30 m × 0.32 mm × 5 µm). Detailed information about the working conditions of the applied TD-GC-FID system can be found elsewhere [27]. The identification of chemical compounds that were emitted most intensively was performed with the use of the gas chromatography coupled with mass spectrometry (GC-MS) system (GC Agilent Technologies 6890 and 5873 Network Mass Selective Detector; Agilent Technologies, Agilent, Santa Clara, CA, USA). Previously mentioned samples were treated in the µ-CTE250 system for 10 min at 100 °C under the constant flow rate. After the sampling process (using the sorption medium specified earlier), the analytes were liberated using TD in similar conditions as for the previously mentioned TD-GC-FID system. In brief, working parameters of applied GC-MS system were as follows: GC capillary column (J&W DB-5MS) of 60 m × 0.25 mm × 1 μm, helium constant gas flow rate of 1.5 mL∙min^−1^, GC-MS transfer line temperature of 280 °C, quadrupole MS analyzer temperature of 150 °C, and MS ion source temperature of 250 °C. Before every sampling period, the chambers with the glass Petri dish inlet were baked out at an elevated temperature, and the background value was investigated. Detailed information about the equipment characteristics, preparation protocol, and the analytical procedure conditions is provided elsewhere [28].

An integrated measuring instrument (Metriso-2000 ohmmeter; GMC-I Messtechnik GmbH, Nürnberg, Germany) with the electrode system was used in the study to determine electrostatic properties following PN-EN 61340-2-3. This method of testing surface resistance is used to assess the risk associated with the threat of static electricity. The electrode system is a measuring electrode with two coaxially aligned electrodes, namely cylindrical and ring electrodes. A measurement range from 1 × 10^3^ Ω to 1 × 10^12^ Ω is sufficient for testing the resistance of the modified electrostatic POM material used in this study. According to the standard, measurements are performed on the plate-shaped specimens with dimensions of 120 mm × 80 mm. However, this also allows the analysis of the final products, so the resulting filaments were analyzed in this case.

The contact angle of the samples was measured on an MWD workshop optical microscope with a DSA 10 MK2 Drop Shape Analysis System (Krüss Optronic, Hamburg, Germany) with demineralized water. The sample was leveled on the measuring table. Then, the measuring drops were applied to the sample surface with a micropipette. The shape of the drop was observed with a microscope, while the height and radius of the drop’s contact surface were measured using the goniometer heads and the sliding table. For each sample, ten measurements were taken.

The samples for mechanical tests were made following the standard PN-EN/ISO 527-2:2012. Tensile tests were performed using the Instron 4465H 1937 tensile testing machine (Instron, Norwood, CO, USA) with elongation head and an extensometer. Tensile tests were performed at a constant speed of 1 mm/min (for Young’s modulus) and 50 mm/min (tensile strength and elongation at break). Reported values are the average of at least five measurements.

Differential scanning calorimetry (DSC) analyses were performed on a Mettler Toledo TGA/DSC 1 (Mettler-Toledo AB, Stockholm, Sweden) equipped with a cooling system. Prepared samples of 6–7 mg were introduced into aluminum pans and heated from 50 to 240 °C, held for 2 min, then cooled to 50 °C. Samples were then reheated to 270 °C, followed by a second crystallization. Heating and cooling rates were 10 °C/min. All DSC scans were performed under a N_2_ atmosphere. A nitrogen flow of 60 mL/min was maintained throughout the test. Baseline corrections were also done with the supplied software.

The thermogravimetric analysis (TGA) was performed on a Mettler Toledo TGA/DSC 1 (Mettler-Toledo AB, Stockholm, Sweden). Synthetic air was used as a purge gas in this method at a set flow rate of 60 mL/min. Samples of 22 ± 1 mg were loaded into ceramic pans to evaluate loss in mass and to determinate the thermal stability of obtained polymer mix. The climatic chamber was set at 50 °C then heated to 900 °C at a heating rate of 10 °C/min.

The dogbone ISO 527-2-1A specimens were imaged using X-ray microcomputed tomography (μCT) (v|tome|x s, GE Sensing & Inspection Technologies, phoenix|x-ray, Wunstorf, Germany). The samples were placed on the polymer stand and scanned at 180 kV with a current of 2000 μA. One thousand scans of each sample were obtained at a total scan time of 150 s—the established scan parameters allowed to register an optimal contrast of the image with 10 μm resolution. To identify changes in the microstructure of the analyzed samples, the acquisition of micro-CT projections was carried out in an 8-bit greyscale. Image acquisition was carried out using the micro-CT system (GE Sensing & Inspection Technologies, Wunstorf, Germany), providing a sequence of 2D images. The reconstruction and visualization were conducted using VGStudio MAX 2.1 software (Volume Graphics, GmbH., Heidelberg, Germany).

Each material was dried in laboratory dryer for 8 h at 60 °C, then printed on a scientific test FDM 3D printer (University of Silesia in Katowice, Katowice, Poland) and a Blixet B50-multi FDM 3D printer (Blixet Sp. z o.o., Sosnowiec, Poland) with closed printing chamber and build size of 400 mm × 400 mm × 520 mm from STL files with GCODE generated on Repetier Host with Slic3r slicing software (1.3.1-dev, Open-source platform) with the usage of Dimafix pen glue adhesive. Parameters of 3D printing are presented in Table 3.

## 3. Results and Discussion

### 3.1. Headspace Analysis

**Figure 2:**
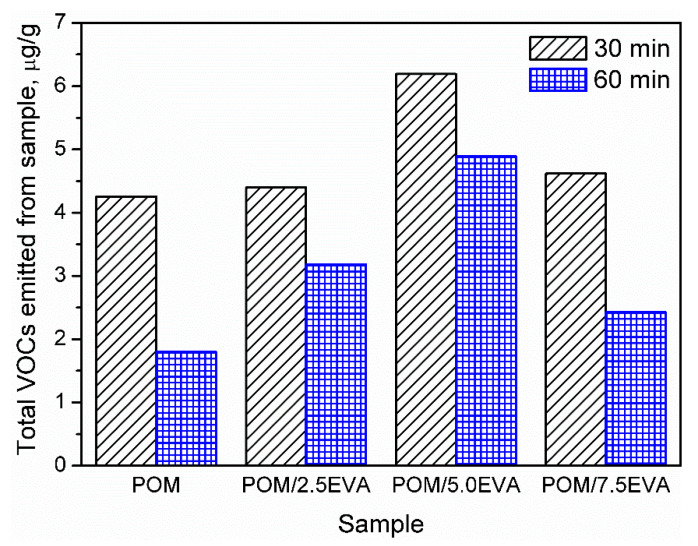
Values of the total volatile organic compounds (TVOCs) parameters for investigated polyoxymethylene (POM)/ethylene-vinyl acetate (EVA) blends.

Determination of VOC emissions from different materials is crucial for human safety and the development of the least harmful products. However, this aspect of polymeric materials is not very often investigated and described in the literature. In the case of polymeric materials, emissions during materials’ processing are more commonly analyzed [29]. On the other hand, emissions during processing are often related to the changes in the chemical structure of the material, which can result in emissions during the further stages: postproduction treatment, packing, transport, and use of various products. Therefore, to fully evaluate the benefits and drawbacks of the product, it is essential to determine its impact on the environment and human health during use, when the humans’ contact with the product is the most intensive [30]. Such an approach is more and more pronounced these days. In the presented case of POM and EVA materials, which are intended to be used in 3D printing, this is especially important, because of the growing popularity and accessibility of 3D printers, not only for industrial but also for hobby and household applications [31]. Generally, recently conducted studies of Noguchi and Yamasaki [32] indicate that both POM and EVA are characterized by relatively low VOC emission rates in comparison to polyolefins or, especially, poly(vinyl chloride). However, these authors conducted their analysis at 50 °C and did not investigate emitted VOCs in qualitative terms, as is done in the present work. Our results are summarized in Figure 2 and Table 4, where emission is shown quantitatively and qualitatively.

It can be seen that the volume of emission strongly depends on the content of EVA. Such an effect is associated with higher emissions related to neat EVA when compared to POM [32]. The highest emissions were observed for 5.0 wt.% content of EVA. However, for all contents, emissions are relatively at similar levels. Moreover, values of the TVOCs parameter obtained after two times of sample conditioning are presented in Figure 2. Before the analysis, samples present in chambers were purged with gas to stabilize the analysis conditions. It can be seen that the conditioning of samples for a longer time significantly decreases the emission level, which indicates that the majority of VOCs are emitted during the first 30 min of analysis. Such an effect can be exciting and essential for the potential applications of analyzed materials because proper postproduction treatment of the product may significantly reduce its environmental impact. Considering the more and more pronounced issues of the environmental aspects of different processes and products, the influence of sample conditioning on the VOC emission should be investigated in further studies.

Table 4 presents the main VOCs detected during analysis of the prepared materials. They are grouped according to their chemical structure. Regarding POM, literature reports related to the VOCs emitted by this material are mainly focused on the most abundant compound—formaldehyde [29,33,34]. However, this compound was not detected here because of the application-defined type of sample-treating instrument and the kind of sorption material. The application of Tenax TA as a sorption material gives a possibility to collect the analytes from the VOCs group, from C_5_ even up to C_30_.

In consequence, there is a very low possibility that any formaldehyde potentially emitted from the studied samples will be adsorbed and collected by applied type of sorption medium. Besides, the application of the GC-FID system and defined type of GC column gives a possibility to perform the analysis of a broad spectrum of VOCs, excluding formaldehyde. The TD-GC system is not a suitable configuration for the formaldehyde analysis. Generally, in the analytical laboratory practice for formaldehyde emission studies, the containers filled with silica gel, cellulose, or glass fiber filters coated with appropriate reagent, namely 2,4-DNPH (2,4-dinitrophenylhydrazine), are applied. Then, the derivatization product (2,4-dinitrophenylhydrazine derivative of formaldehyde) is analyzed with the use of high-performance liquid chromatography (HPLC) combined with a UV-Vis detector [35].

It can be seen that significant traces of hydrocarbons, alcohols, phenols, aldehydes, and ketones, as well as carboxylic acids and esters, were emitted from the samples. Such a combination of organic compounds is typical for the headspace analysis of polymeric materials [30]. For EVA materials, a similar composition of VOCs was already reported for SAFC Biosciences EVA BIOEAZE bags [36]. The presence of hydrocarbons is related to the polymeric nature of materials. These compounds are often detected, as confirmed by other works [32,37]. The presence of other compounds mentioned above is often associated with the processing of the material and even minimal changes in chemical structure during thermal and mechanical treatment, e.g., extrusion. Although polymeric materials are usually processed at temperatures noticeably below their onset of degradation, they are still subjected to oxidation. In the case of POM, even at 160 °C, slight decomposition may occur, resulting in the generation of aldehydes, ketones, or alcohols [38]. For EVA, the most commonly reported compounds are those containing hydroxyl and carbonyl functional groups, such as alcohols, lactones, esters, and ketones [39]. Barlow et al. [37] detected a significant amount of hydrocarbons, of which over 80% were characterized as heavy hydrocarbons. Among other groups, they noted the presence of acetaldehyde, benzaldehyde, acetone, and formic and acetic acids. These results correspond with the data presented in Table 4. Except for these compounds, related to the use of POM and EVA, other chemicals were detected, whose presence was ascribed to the storage and processing of various polymeric materials in the same laboratory hall. The presence of low amounts of toluene, styrene, and trichloromethane was noted and was not mentioned here.

Table 4 also presents areas under the peaks related to the included compounds; however, in this case, these should not be directly used to determine the specific amounts of particular emissions quantitatively. Nevertheless, changes in signals’ intensity still can suggest an increase or decrease of specific emissions. With the addition of EVA, a significant increase in the intensity was noted for peaks ascribed to the presence of vinyl compounds, acetic and formic acids, acetone, and butylated hydroxytoluene (BHT). Except for the last one, the presence of other compounds is associated with the chemical structure of EVA and was previously noted by Barlow et al. [37]. BHT is commonly applied as an antioxidant for polymers, e.g., for polyolefins, and was also detected by headspace analysis of PP and PE in another work [40].

Regarding hydrocarbons, the intensity of signals was quite similar; however, in total, the area under peaks related to presented hydrocarbons slightly decreased with EVA content. For alcohols, phenols, and aldehydes, the decrease or absence of signals associated with a particular compound was followed by the increase or appearance of other ones. In the case of phenols, signals for BHT were weakened with the increase of the EVA content, but they were substituted by 6-*tert*-butyl-2,4-xylenol, the compound used as polymerization inhibitor [41]. The primary ketone detected for neat POM was acetophenone. When POM was partially substituted by EVA, acetophenone was still present, but its amount was noticeably lowered, and it was replaced with other ketones. Therefore, the total area under peaks ascribed to the presence of ketones was higher when EVA was added. Generally, these differences are associated with the different composition of the material and hence also the different products of thermo-oxidative decomposition, which may partly occur during thermomechanical processing with extruders or injection molding machines.

Values of vapor pressure, which can be considered as the quantitative measure of a material’s volatility, are presented for all compounds. In general, there is no actual value of vapor pressure that determines if the compound is considered to be a VOC [42]. Although it is not a strict definition or standard, the United States Environmental Protection Agency exempts solvents in consumer products with a maximum vapor pressure of 13.3 Pa at 20 °C. These compounds show little or no volatility, which would not result in significant VOC emissions [43]. According to these criteria, part of the compounds listed in Table 4 should not be considered as VOCs. Nevertheless, for the full assessment of the harmful effects of chemicals, except the volatility, the exposure limits should also be considered. A combination of these factors would provide a broader view of the threat posed by particular materials.

Therefore, except for the vapor pressure, Table 4 presents codes according to NFPA 704 (Standard System for the Identification of the Hazards of Materials for Emergency Response), which is commonly used for classification of chemicals by the risk they pose towards human health and safety [44]. The NFPA 704 system is commonly known as the “safety square” or “fire diamond” and is applied to quickly and easily identify the risks and select the proper procedure for handling chemicals. It was developed by the National Fire Protection Association from the United States. It assesses chemicals’ flammability, health risk, and instability (noted as F, H and I in Table 4) on a scale from 0 (minimal hazard) to 4 (severe hazard), also providing information about special hazards related to the material.

It can be seen that a couple compounds are listed whose health hazard (blue) indicator is at level 3, meaning that short exposure could cause serious temporary or moderate residual injury. Among these compounds, limonene, acetic acid, and formic acid should be considered as a significant threat, especially considering their values of vapor pressure. On the other hand, they are also characterized by the flammability rating (red) of 2, meaning that they must be moderately heated or exposed to relatively high ambient temperature before ignition can occur. Such compounds are characterized by the flash point values between 37.8 and 93.3 °C.

### 3.2. Melt Flow Index

Melt flow index (MFI) is a measure of output rate of polymers in grams going through a standard die with fixed pressure being applied to the melt at a particular temperature. Therefore, it is a measure of material flowability, which is an essential parameter for 3D printing. It is an assessment of average molecular mass and is an inverse measure of the melt viscosity; in other words, the higher an MFI, the more polymer flows under test conditions [45]. Wang et al. [46] indicated that the MFI value of 10 g/10 min would be suitable for fast and straightforward printing. However, their research was associated with PLA filaments. Nevertheless, such a value guarantees the proper flow of material during printing. However, it dies not cause the “spilling” of the printed specimen. At the same time, it is essential to remember that MFI analysis should be supplemented by other investigations to fully assess the material’s potential for 3D printing [46]. Table 5 presents MFI values for the applied POM and EVA materials and their blends.

### 3.3. Mechanical Performance

Mechanical properties of raw materials, as well as POM/EVA blends, are presented in Table 6. It can be seen that the mechanical performance of blends is determined by their composition, since POM and EVA differ significantly. As mentioned above, in terms of mechanical performance, the modification of POM was intended to overcome its drawbacks and provide it with some features of EVA, e.g., lower stiffness and shrinkage.

The tensile strength of POM/EVA blends decreased with the increase of EVA content. The addition of 2.5 wt.% of EVA caused an 11.5% reduction in tensile strength, followed by 15.8 and 20.8% drops for 5.0 and 7.5 wt.% of the modifier, respectively. Simultaneously, elongation at break increased. Similar results for compatibilization of POM with EVA were observed by Uthaman et al. [24]. A combination of these two properties, expressed by the area under stress–strain curves, can be used to assess the toughness of the material. Its integration results in the quantification of energy that can be withstood by the material before its failure [47]. Ideally, tough material should be strong and ductile at the same time, so it should be able to resist the highest stress possible for the highest elongation possible. Table 6 also presents values of materials’ toughness. The addition of EVA to POM resulted in the increase of toughness, which was mainly affected by increasing elongation at break. Such an effect can be considered very beneficial for the application of the investigated materials in 3D printing.

### 3.4. Surface Resistance

**Figure 3:**
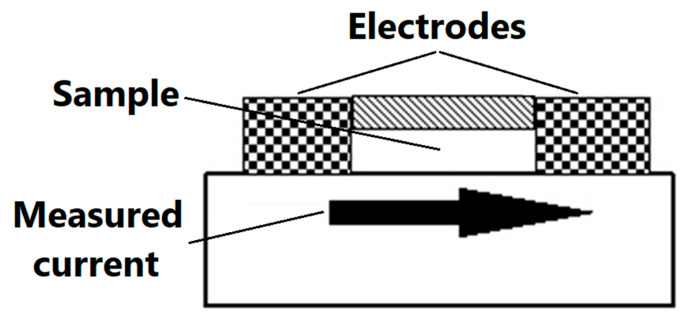
Scheme of the measurement of surface resistance.

The purpose of electrostatic measurements is to determine the characteristics of the tested object, namely the state (degree) of electrification or antistatic properties. Both of these features are associated with the intensity of the electric field, which is the fundamental parameter used to describe the electric field surrounding electrostatic charges. Electrical resistance is defined as the quotient of DC voltage and current. The object resistance is the quotient of the DC voltage applied to the tested object and the current flowing between the electrodes through the tested object. Resistance results from the relationship described by Ohm’s law. The surface resistance is the electrical resistance of the material expressed in ohms, measured between the measuring electrodes on the same surface of the material being tested (as shown in Figure 3)—following relevant standards [48].

The samples of 3D printer filament were used to form a surface corresponding to the minimum standard requirements. The results of the performed analysis are presented in Table 7. Polyoxymethylene (POM) copolymer Tarnoform 300 EC2 antistatic and electrically conductive injection molding grade material showed a resistance of 1.75 × 10^4^ Ω. The addition of EVA copolymer resulted in a slight increase in electrical surface resistance. Such an effect was associated with the significantly higher value of this parameter for neat EVA—3.63 × 10^15^ Ω.

### 3.5. Contact Angle

According to obtained results, POM is relatively close to the hydrophobic polymer, with a static contact angle in the range of 82–85°, but still fits into a hydrophilic surface. According to Law [49], because the changes in wetting and adhesion interaction are gradual, there is no cutoff to be found at 90° for static contact angle (θ), advancing contact angle (θ_A_), and receding contact angle (θ_R_). In the case of future applications, hydrophilic nature ensures better surface adhesion while, on the other hand, the hydrophobic nature of the surfaces helps to prevent self-cleaning and stickiness. In Table 8, values of the contact angle measured for the prepared samples are presented.

Other studies [50] related to EVA containing 18 wt.% of vinyl acetate show that the raw material has a water contact angle of around 68°. From the results obtained, it can be concluded that the addition of EVA copolymer to POM in this dose does not have a significant impact on the contact angle parameter.

### 3.6. Thermal Properties

**Figure 4:**
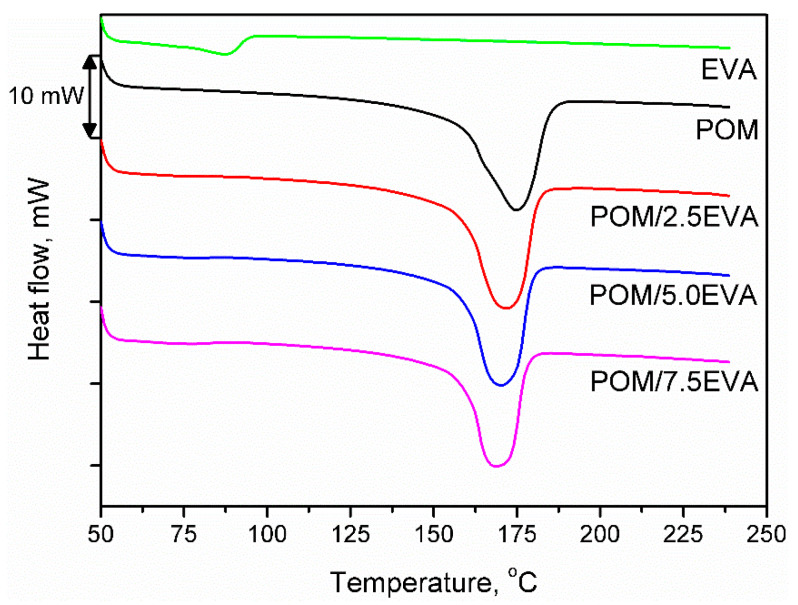
DSC thermograms for prepared samples.

Figure 4 shows the DSC curves obtained during the second heating of the samples. It was noted that despite the significant differences in melting temperatures of raw materials, the addition of EVA to POM did not significantly affect the melting point. The onset was determined to be around 158–159 °C in all cases, while the maximum melting peak was recorded in the range of 168–175 °C. With the increase of EVA content, melting temperature (T_m_) was shifted from initial 174.75 °C to 168.70 °C for 7.5 wt.% addition of EVA, through 171.66 and 170.19 °C for 2.5 and 5.0 wt.%, respectively. A similar effect was noted in other works [51]. Enthalpy of melting was decreasing with the increase of EVA content and was in the range of −92.97 to −101.30 J/g. For 3D printing, the performed modification of POM should not affect the printing parameters. The only possible change may be a slight lowering of the printing temperature, which may affect the solidification of material and adhesion of the print to the table.

### 3.7. Thermal Stability

**Figure 5:**
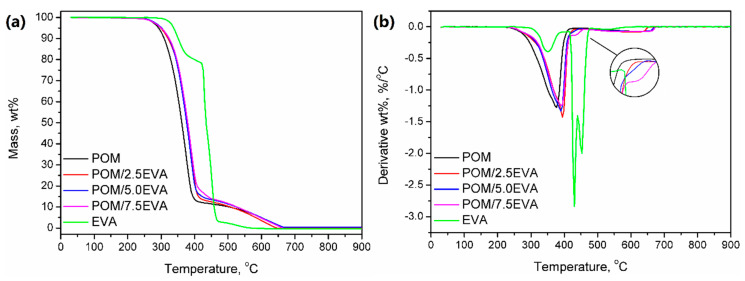
(**a**) Thermal stability and (**b**) differential thermogravimetric curves for prepared samples.

In Figure 5, results of the thermogravimetric analysis of the prepared materials are presented. It can be seen that both raw materials show different thermal stabilities, so the modification of POM with EVA should enhance this parameter. For neat POM, thermal decomposition starts (determined by the temperature at 2 wt.% mass loss) at 271.7 °C, while it starts at 323.5 °C for EVA. Therefore, modification of POM shifted this parameter to higher temperatures—from 273.4 to 276.2 °C. Typically, when TGA analysis is performed under the nitrogen atmosphere, POM shows a one-step decomposition ending around 400 °C [52]. However, in the presented study, the analysis was performed under a synthetic air atmosphere in order to imitate the conditions that occur during 3D printing. As a result, the course of decomposition was changed above 400 °C, which was associated with the thermolysis of degradation products generated at lower temperatures.

The curve observed for thermal decomposition of EVA is typical for this material; similar results, indicating three-step decomposition, were observed by other researchers [53]. The first step is associated with the degradation of the vinyl acetate phase and results in the generation of acetic acid. The second step is related to the chain scission of the ethylene phase. The values of mass loss for these steps are associated with the vinyl acetate content in EVA. In Figure 5b, it can be seen that modification of POM with EVA and presence of the ethylene phase results in the slight change of decomposition course, which is more pronounced for rising EVA content. The third step, observed at temperatures over 465 °C, is due to conditions of analysis, particularly the application of synthetic air as atmosphere gas [53].

### 3.8. Quantitative Microcomputed Tomography Scanning

**Figure 6:**
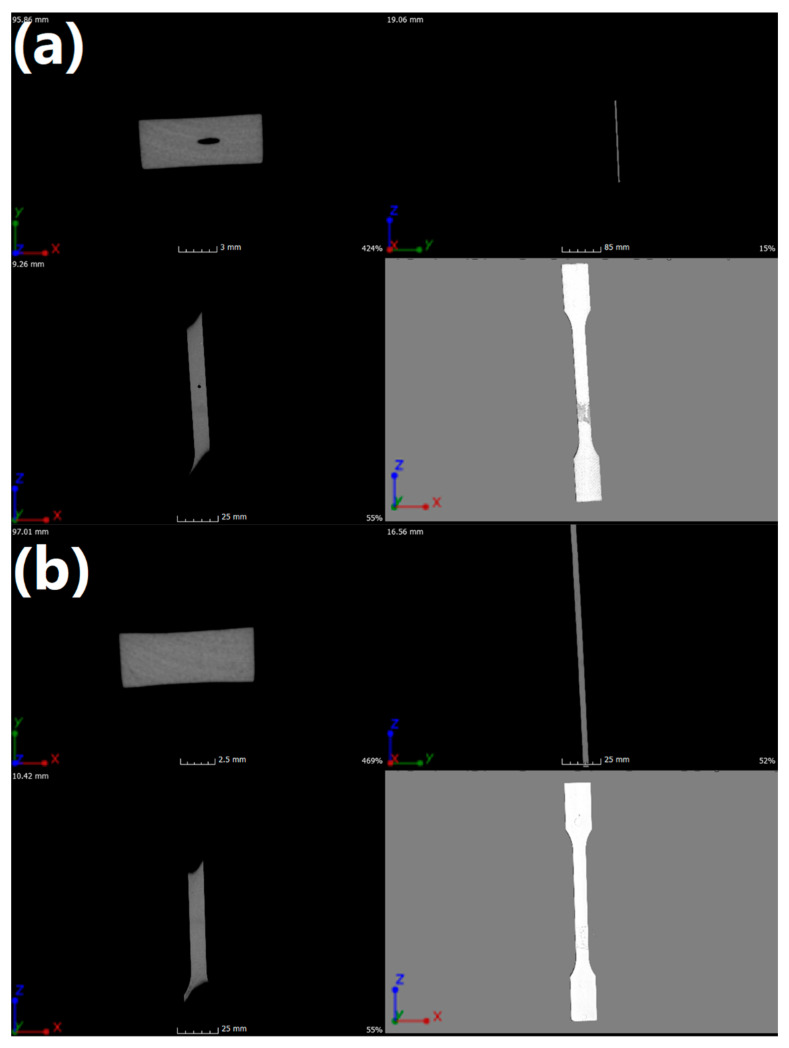
Images of (**a**) neat POM and (**b**) POM/5.0EVA samples obtained using µCT.

To reveal the heterogeneity of the material and the defects arising in the injection process, internal structure tests were performed using µCT. The results of the performed analysis can be seen in Figure 6 and Table 9. In Figure 6, X, Y, and Z indicate frontal, longitudinal, and sagittal axes, respectively. Imaging was performed to show changes in density as well as changes in material continuity and possible errors in the plasticization process. By analyzing obtained images, it was found that there were defects, expressed by the lower density of the material, probably resulting from injection channels that were too long and associated with excessive duration of flow for neat POM. The grey stain on the surface of the POM sample resulted from the gluing of the sample to the table.

In the case of the POM/5.0EVA sample, presented in Figure 6b, no discontinuity of material was detected, which would indicate the presence of defects. This may indicate a better linear flow rate due to the addition of EVA. Injection parameters for all manufactured details were identical. The parameters have not been changed intentionally so as not to introduce further variables, as this could introduce additional defects in the injection process and prevent them from being assigned to a specific factor or indicate the etiology of their creation due to the presence of too many variables in one experiment. Moreover, the homogeneity of the sample points to the good interfacial compatibility.

None of the analyzed details showed standard defects resulting from the use of an improper plasticization process or a lack of compatibility between individual components of the injected blends.

### 3.9. Suitability for 3D Printing

**Figure 7:**
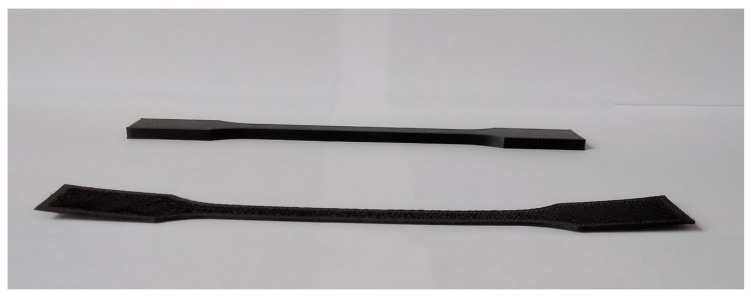
Warping of neat POM at the beginning of the 3D printing compared to the completed specimen.

The extruded 3D-printing filament with a diameter beyond the tolerance range of 2.85 ± 0.05 mm due to the limitation of the twin-screw extruder and original filament collection system was printed on modified 3D printers, allowing the use of a wide range of diameters with reduced print quality. ISO 527-2-1A specimens were printed with each material ten times. The results of the performed tests are presented in Table 10 and Figure 7.

From the beginning of the printing process, the 3D printer operator was waiting for any sign of print detachment from the printing bed. If the problem occurred, the process was stopped and considered as unsuccessful. Each research material was used in at least one attempt to complete an unsuccessful print in order to check for adhesion to the printing table, where despite the peeling problem it was allowed to finish the printing process.

The main problem with printing with this material was insufficient adhesion to the printing table despite using glue adhesive. Most of the printed specimens started to warp at the beginning of the printing process, like POM without any addition, which is shown in Figure 7 in comparison to the completed 3D-printed specimen.

Specimens with 7.5% addition of elastic EVA exhibited low filament column strength. This problem caused bending and buckling of the filament before plasticization of the material in the printing nozzle.

The addition of EVA improved material adhesion to printing bed and resulted in higher prints before warping, in addition to allowing some printings to finish without any deformation. Such an effect can be related to the lower shrinkage and higher flexibility of the EVA phase in comparison to the POM material. Incorporation of 5.0 and 7.5 wt.% EVA resulted in a similar success rate. As a result, these concentrations of modifier may be considered as practically equally suitable for use, depending on the desired properties of the final print (such as those related to the mechanical performance). Nevertheless, further works should examine the impact of the temperature of the printing table and the type of glue used to provide adhesion between print and table.

**Figure 8:**
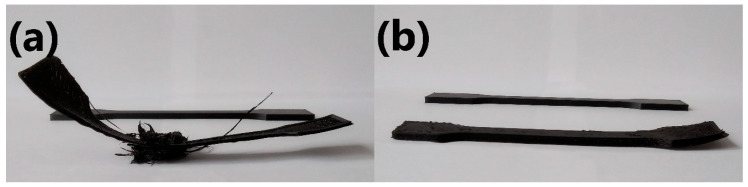
Unsuccessful and successful 3D-printed specimens of (**a**) POM/2.5EVA and (**b**) POM/5.0EVA.

Figure 8 shows unsuccessful attempts to print ISO-527 specimens with the addition of 2.5 and 5.0 wt.% of EVA in comparison to one of the successful prints.

## 4. Conclusions

This study aimed to investigate the properties of polyoxymethylene modified with 2.5, 5.0, and 7.5 wt.% of ethylene-vinyl acetate and determine its potential for applications in fused deposition modeling. Modifications were performed to overcome the issues limiting POM’s use in 3D printing, namely those related to shrinkage and warping of specimens. Simultaneously, other aspects of these materials, were investigated namely the mechanical performance, the thermal stability, and the very rarely analyzed emissions of volatile organic compounds.

It was noted that the performed modifications did not significantly affect the amount of volatile organic compounds emitted from materials, with the exception of the sample containing 5.0 wt.% of EVA. The composition of emitted VOCs slightly differed between particular composition variants, which was related to changes in the chemical composition of the material. Nevertheless, we strongly believe that further studies related to 3D printing should include the analysis of volatile organic compounds emitted during printing and released from the final products, especially since the FDM technique is gaining popularity as a hobby for many people.

Moreover, modification of POM, considered as an engineering polymer with EVA, resulted in reduced stiffness and strength of the material, simultaneously enhancing its ductility, which in turn increased the toughness by more than 50% for 7.5 wt.% EVA loading. Regarding thermal properties, as well as the surface resistance of the material, the performed modifications caused only slight changes, which should not affect the potential application of the material in 3D printing. Melting temperature was slightly shifted from the initial 174.75 °C to 168.70 °C for 7.5 wt.% addition of EVA, which could enable the slight reduction of nozzle temperature. The thermal stability of the material was maintained at a similar level and even slightly enhanced. The onset of the decomposition exceeding 275 °C guarantees a safe processing window for 3D printing, which enables the use of analyzed materials without their decomposition.

Quantitative microcomputed tomography scanning has shown that the addition of EVA caused a better linear flow rate or allowed a better selection of injection parameters for injection molding samples. At the same time, no evidence of defects resulting from improper plasticization or incompatibility between individual components of the injected blends was found. The printing process on two different printers has shown that the addition of EVA copolymer increased the possibility of a successful print of an ISO 527-2-1A standardized specimen, giving space for further development. Loadings of EVA at 5.0 and 7.5 wt.% could be considered as practically equally suitable for use, depending on the desired properties of the final prints. Further research studies should be more focused on the analysis of the overall influence of EVA addition on the printability of POM/EVA blends. An approach consisting of proper peeling tests would enable a better understanding of the usefulness of POM-based materials in incremental digital techniques not only limited to FDM 3D fabrication. Modifications of the mechanical and structural parameters of POM by the incorporation of EVA could allow it to be used in additional applications, e.g., in 4D printing, due to the higher flexibility of the material.

## Figures and Tables

**Table 1:** Parameters of the extrusion for particular samples.

Sample	Screw Speed, rpm	the Temperature in Extruder Zones, °C				Pressure, Bar
		Z1–Z2 ^1^	Z3–Z6 ^1^	Z7–Z8 ^1^	Z9, Z10, N1, N2 ^1^	
POM	200	190	170	180	200	90
POM/2.5EVA	200	190	170	180	200	87
POM/5.0EVA	200	190	170	180	200	86
POM/7.5EVA	200	190	170	180	200	84

^1^ Z1–Z10, heating/liquid cooling zones; N1 and N2, extrusion-heating-only zone.

**Table 2:** Parameters of the injection molding of investigated samples.

Parameter	Value
Material temperature, °C	230 ± 2
Mold temperature, °C	30 ± 1
Injection speed, mm/s	25
Cycle time, s	60
Injection pressure, bar	420
Press pressure, bar	350

**Table 3:** Parameters of 3D printing of investigated samples.

Printing Parameter	Value
Nozzle diameter, mm	0.4
Layer height, mm	0.1
Fill pattern	Rectilinear
Fill percentage, %	100
First layer temperature, °C	230
Other layers temperature, °C	230
Bed temperature, °C	120
Chamber temperature, °C	60
Air humidity, %	35
Number of layers	40

**Table 4:** Chemical compounds detected by headspace analysis of investigated POM/EVA blends and neat POM.

Detected Compound	Chemical Formula	Chemical Structure	Vapor Pressure, Pa	NFPA 704			Area, %			
				H	F	I	POM	POM/2.5EVA	POM/5.0EVA	POM/7.5EVA
*Hydrocarbons*	*CxHy*									
Limonene	C_10_H_16_	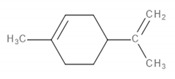	190	3	2	0	0.42	1.08	0.60	1.45
Tridecane	C_13_H_28_	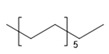	5	1	2	0	2.46	1.42	2.05	1.04
Pentadecane	C_15_H_32_	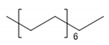	0.4	1	1	0	0.73	0.52	0.61	0.47
Hexadecane	C_16_H_34_	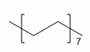	0.2	1	1	0	1.30	0.68	0.61	0.61
Eicosane	C_2_0H_42_	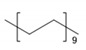	6·× 10^−4^	0	1	0	0.58	0.63	0.35	0.42
*Alcohols*	*CxHyOH*									
Cyclohexanol	C_6_H_12_O	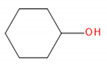	133	1	2	0	-	0.99	1.21	1.02
2-Ethyl-1-hexanol	C_8_H_18_O	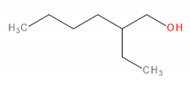	30	2	2	0	0.71	0.54	0.54	0.47
2-Phenyl-2-propanol	C_9_H_12_O	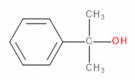	69^at 37.8 °C^	2	2	0	4.86	4.83	3.94	3.64
*Phenols*	*ArOH*									
6-*tert*-Butyl-2,4-xylenol	C_12_H_18_O	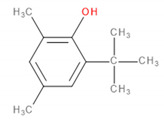	5.3	3	1	0	5.65	3.21	2.30	2.60
Butylated hydroxytoluene	C_15_H_24_O	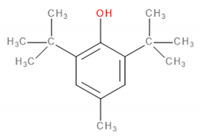	1.3	2	1	0	3.50	6.64	5.36	7.78
*Aldehydes*	*CxHyCHO*									
Benzaldehyde	C_7_H_6_O	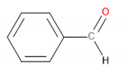	133	2	2	0	0.99	0.84	0.89	1.60
Nonanal	C_9_H_18_O	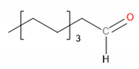	49	2	2	0	2.21	1.92	1.62	1.63
Decanal	C_10_H_20_O	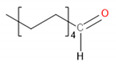	13	1	2	0	1.98	1.73	1.71	1.56
*Ketones*	*CxHyCO*									
Acetone	C_3_H_6_O	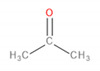	30,600	1	3	0	-	0.69	0.69	0.92
Acetophenone	C_8_H_8_O	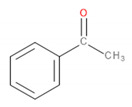	52	2	2	0	18.01	15.88	10.99	12.16
2-Decanone	C_10_H_20_O	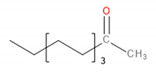	33	0	2	0	-	4.29	3.33	4.44
3-Decen-2-one	C_10_H_18_O	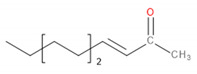	28	No data			-	0.72	0.83	0.53
2-Dodecanone	C_12_H_24_O	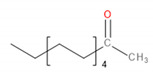	2.7	No data			-	3.65	4.41	3.31
2,6-Di-*tert*-butylquinone	C_14_H_20_O_2_	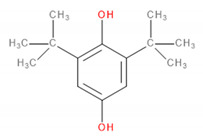	1.3	2	1	0	1.12	0.98	1.04	0.95
2-Tetradecanone	C_14_H_28_O	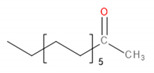	0.9	0	2	0	-	1.84	2.12	1.99
*Carboxylic acids*	*CxHyCOOH*									
Acetic acid	C_2_H_4_O_2_	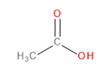	1520	3	2	0	1.48	6.08	8.44	9.87
Formic acid	CH_2_O_2_	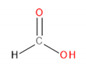	4666	3	2	0	-	1.09	0.84	1.22
*Esters*	*CxHyCOOCxHy*									
Vinyl caprylate	C_10_H_18_O_2_	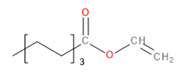	30	0	2	0	-	0.95	1.02	0.97
2-Oxepanone	C_6_H_10_O_2_	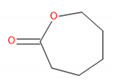	1.3	2	0	0	5.22	5.07	4.07	6.11
Vinyl decanoate	C_12_H_22_O_2_	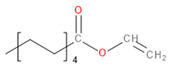	4.4	0	1	0	-	1.33	1.53	1.41

**Table 5:** Values of the melt flow index for investigated samples.

Sample	MFI, g/10 min
POM	4.04
POM/2.5EVA	2.15
POM/5.0EVA	2.30
POM/7.5EVA	3.38

**Table 6:** Mechanical properties of prepared materials.

Material	Tensile Strength, MPa	Elongation at Break, %	Young’s Modulus, MPa	Toughness, J/cm^3^
POM	50.0 ± 1.0	5.7 ± 0.4	2812 ± 104	201 ± 11
POM/2.5EVA	44.2 ± 0.2	8.6 ± 0.3	2094 ± 74	283 ± 19
POM/5.0EVA	42.1 ± 0.7	8.6 ± 0.1	2047 ± 71	275 ± 2
POM/7.5EVA	39.6 ± 0.9	9.4 ± 0.3	1902 ± 46	303 ± 16
EVA	9.9 ± 0.6	354.9 ± 13.8	26 ± 1	961 ± 27

**Table 7:** Values of surface resistance for POM and POM/EVA blends.

Sample	Surface Resistance, 10^4^ Ω
POM	1.75 ± 0.89
POM/2.5EVA	1.89 ± 0.78
POM/5.0EVA	2.09 ± 0.89
POM/7.5EVA	2.11 ± 1.27

**Table 8:** Values of contact angle for prepared samples.

Material	Water Contact Angle, °
POM	82.90 ± 1.47
POM/2.5EVA	85.00 ± 0.96
POM/5.0EVA	82.10 ± 2.04
POM/7.5EVA	84.30 ± 0.67

**Table 9:** The number of defects detected for analyzed samples.

Sample Name	Number of Defects
POM	26
POM/2.5EVA	0
POM/5.0EVA	0
POM/7.5EVA	0

**Table 10:** Results of 3D printing attempts.

Material	Outcome UoS Printer	Outcome Blixet B-50 Printer
POM	1/10 succeed	2/10 succeed
POM/2.5EVA	3/10 succeed	3/10 succeed
POM/5.0EVA	5/10 succeed	4/10 succeed
POM/7.5EVA	4/10 succeed	4/10 succeed
